# Cortisol-Induced Masculinization: Does Thermal Stress Affect Gonadal Fate in Pejerrey, a Teleost Fish with Temperature-Dependent Sex Determination?

**DOI:** 10.1371/journal.pone.0006548

**Published:** 2009-08-07

**Authors:** Ricardo S. Hattori, Juan I. Fernandino, Ai Kishii, Hiroyuki Kimura, Tomomi Kinno, Miho Oura, Gustavo M. Somoza, Masashi Yokota, Carlos A. Strüssmann, Seiichi Watanabe

**Affiliations:** 1 Graduate School of Marine Science and Technology, Tokyo University of Marine Science and Technology, Tokyo, Japan; 2 Laboratorio de Ictiofisiología y Acuicultura, Instituto de Investigaciones Biotecnológicas, Instituto Tecnológico de Chascomús, Chascomús, Argentina; Temasek Life Sciences Laboratory, Singapore

## Abstract

**Background:**

Gonadal fate in many reptiles, fish, and amphibians is modulated by the temperature experienced during a critical period early in life (temperature-dependent sex determination; TSD). Several molecular processes involved in TSD have been described but how the animals “sense” environmental temperature remains unknown. We examined whether the stress-related hormone cortisol mediates between temperature and sex differentiation of pejerrey, a gonochoristic teleost fish with marked TSD, and the possibility that it involves glucocorticoid receptor- and/or steroid biosynthesis-modulation.

**Methodology/Principal Findings:**

Larvae maintained during the period of gonadal sex differentiation at a masculinizing temperature (29°C; 100% males) consistently had higher cortisol, 11-ketotestoterone (11-KT), and testosterone (T) titres than those at a feminizing temperature (17°C; 100% females). Cortisol-treated animals had elevated 11-KT and T, and showed a typical molecular signature of masculinization including *amh* upregulation, *cyp19a1a* downregulation, and higher incidence of gonadal apoptosis during sex differentiation. Administration of cortisol and a non-metabolizable glucocorticoid receptor (GR) agonist (Dexamethasone) to larvae at a “sexually neutral” temperature (24°C) caused significant increases in the proportion of males.

**Conclusions/Significance:**

Our results suggest a role of cortisol in the masculinization of pejerrey and provide a possible link between stress and testicular differentiation in this gonochoristic TSD species. Cortisol role or roles during TSD of pejerrey seem(s) to involve both androgen biosynthesis- and GR-mediated processes. These findings and recent reports of cortisol effects on sex determination of sequential hermaphroditic fishes, TSD reptiles, and birds provide support to the notion that stress responses might be involved in various forms of environmental sex determination.

## Introduction

In many reptiles, fish, and amphibians, the fate of the differentiating gonad is influenced by a combination of genetic and environmental cues (e.g. pH, relative density, social factors or temperature) early during development [1]–[3]. Strong environmental effects on sex determination characterize environmental sex determination (ESD) as opposed to the strict genetic control of this process which occurs in genotypic sex determination (GSD). The ultimate mechanism(s) of ESD is (are) not very well understood. In temperature-dependent sex determination (TSD), the most extensively studied form of ESD, for example, many molecular and biochemical pathways involved in sex differentiation have been identified, but studies often yield results that suggest the presence of other endogenous modulator(s) of the differentiation process which remain unknown [4]–[9].

One potential endogenous modulator of the process of gonadal sex differentiation in general and of ESD in particular could be stress and the glucocorticoid stress-related hormone known as cortisol. Cortisol has significant roles in metabolism, osmoregulation, growth, and reproduction, and plasmatic levels rise dramatically during stress [10], [11]. It has been also shown that treatment of rainbow trout larvae with cortisol and cortisone inhibited ovarian growth and caused an increase in the proportion of males [12]. More recently, an experimental study showed that exposure of the embryos of two lizard species with TSD to corticosterone caused shifts in sex ratios, although the direction of the shift varied with the species [13]. Likewise, stress (and plasma titres of cortisol) seems to be associated with sex change in sequential (protogynous and protandrous) hermaphroditic species, whereby sex change is cued by social factors (a form of ESD) [14], [15]. These and other studies [16], [17] suggested that stress interferes with the plasma levels of major androgens, especially 11-ketotestosterone (11-KT), because the enzymes involved in their synthesis are also involved in glucocorticoid synthesis and inactivation [15]. In light of these earlier findings and other clues [11], [18], it is surprising that the possibility that stress and cortisol are involved in the sex determination of other species with TSD has not been critically examined nor have the mechanisms of cortisol action on TSD been elucidated.

In this context, we performed a series of experiments to assess the involvement of stress as a modulator of gonadal sex differentiation, and in particular as an agent of masculinization, in the pejerrey *Odontesthes bonariensis*. Temperature has a crucial role in the sex determination of this species since 100% females or males are produced when larvae are exposed to environmentally realistic temperatures (e.g. 17 and 29°C, respectively) during the critical period of sex determination [19], [20]. Sex ratios in natural pejerrey populations vary greatly, sometimes in excess of 80% of one sex, and this variation is likely related to the protracted reproductive season and the wide range of temperatures experienced by the larvae [C.A. Strüssmann, unpublished observations]. Thus, pejerrey has a markedly strong TSD and is an extremely suitable model for the elucidation of the molecular and biochemical mechanisms involved in this mode of sex determination. Experiments were performed to measure the cortisol, 11-ketotestosterone (11-KT) and testosterone (T) concentrations in larvae reared at masculinizing- (MPT), feminizing- (FPT), and mixed-sex-producing temperatures (MixPT), the effects of cortisol and a non-metabolizable glucocorticoid receptor agonist (Dexamethasone) on sex ratio, and of cortisol administration on the sex differentiation molecular cascade. The results provide clear evidence of a relationship between stress (cortisol) and gonadal masculinization in larval pejerrey and suggest that both glucocorticoid receptor- and steroid biosynthesis-mediated processes are involved in the transduction of the thermal environment through stress and ultimately to sex determination.

## Results and Discussion

**Figure 1:**
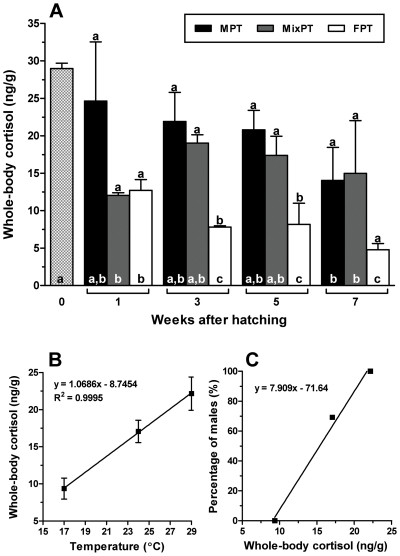
A) Concentration of endogenous cortisol in larvae reared at masculinizing (MPT, 29°C), mixed-sex-producing (MixPT, 24°C), and feminizing (FPT, 17°C) temperatures between 0 and 7 wah. Larvae reared at the MPT and MixPT showed higher cortisol values than those at the FPT between 3 and 5 weeks, a period considered as critical for gonadal sex determination in pejerrey. Different letters inside and above the bars indicate statistically significant differences between weeks for the same temperature and between treatments on the same week, respectively (One-way ANOVA followed by the Tukey test; P≤0.05; GraphPad Prism v.4.00). B) Relation of rearing temperature and the concentration of endogenous cortisol in individual larvae between 1 and 5 weeks after hatching. C) Relation of the mean concentration of endogenous cortisol between 1 and 5 weeks after hatching and the percentage of males.

We first measured whole-body cortisol titres in larvae that were reared at a MPT (29°C), MixPT (24°C) and FPT (17°C) as an indicator of stress at these temperatures during the critical period of sex determination (1 to 5 weeks after hatching, wah) [20]. In this experiment, the final sex ratios for the MPT, MixPT, and FPT groups were 100, 69.2, and 0% males, respectively. These sex ratios, including the relatively high percentage of males at the MixPT, fall within the range reported previously [19], [20]. Cortisol titres seemed to decrease slowly after hatching at the MPT and MixPT but a significant decrease compared to that at the time of hatching was observed only at 7 weeks. In contrast, the decrease was prominent and sustained at the FPT (Fig. 1A). Overall, larvae at the MPT and FPT had the highest and lowest cortisol titres, respectively, with significant (>1.5 fold) differences between the two temperatures between 3 and 5 wah (P<0.05). Cortisol titres in larvae at the MixPT were not statistically different from those at the MPT on a weekly basis (Fig. 1A) but were clearly proportional to temperature at the period which is critical for sex determination (Fig. 1B). In general, cortisol peaks only during the initial phase of the stress response [10], [21] but our results indicate that at the MPT pejerrey larvae seemed to be constantly under the influence of stress. It is interesting to note that a fairly large proportion of the fish at the MixPT in this experiment became males (69.2%), which associated with the relatively high cortisol concentrations observed in this treatment, also supports the causal relation between elevated cortisol and masculinization (Fig. 1C).

**Figure 2:**
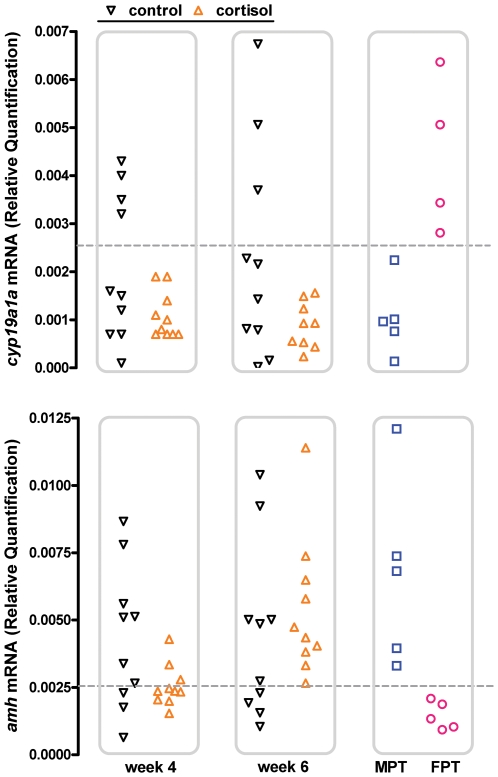
Abundance of mRNA transcripts of the sex differentiation genes *amh* and *cyp19a1a* in cortisol-treated (0.8 mg/g food) larvae at 4 and 6 wah. Typical values obtained at masculinizing (MPT; 7 wah) and feminizing (FPT; 6 wah) temperatures are shown for comparison. Symbols represent individual values for 10 larvae each in control and cortisol-treated groups and 5 larvae each in the MPT and FPT. Values were normalized by the respective values of *β-actin*. Horizontal dotted lines represent threshold values (mean±2SD) for *cyp19a1a* (0.00258) and *amh* (0.00247) that were calculated using values from the MPT and FPT, respectively. Cortisol-treated animals showed consistent *cyp19a1a* downregulation and *amh* upregulation comparable to the typical molecular signature of masculinization of animals at the MPT whereas controls showed a bimodal distribution of mRNA transcripts that agreed with the sex ratio of 69.2% males (that is, 6–7 and 3–4 out of 10 individuals resembled those at the MPT and FPT, respectively).

**Figure 3:**
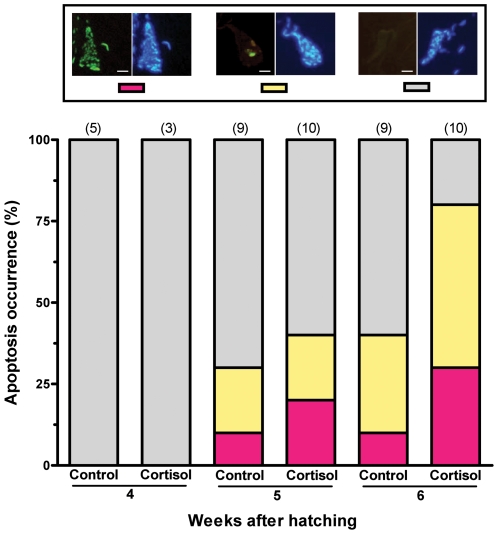
Incidence of gonadal apoptosis (TUNEL assay) in cortisol-treated (0.8 mg/g food) larvae between 4 and 6 wah. Apoptosis occurrence is defined as the percentage of individuals showing each of the categories of apoptosis indicated in the left side of the top panels (red: abundant, yellow: few, grey: none; the right side shows DAPI nuclear staining for visualization of cells). Scale bars represent 10 µm; numbers within parenthesis indicate sample number. Cortisol-treated larvae showed markedly higher incidence of gonadal apoptosis compared to control larvae at 6 wah.

**Figure 4:**
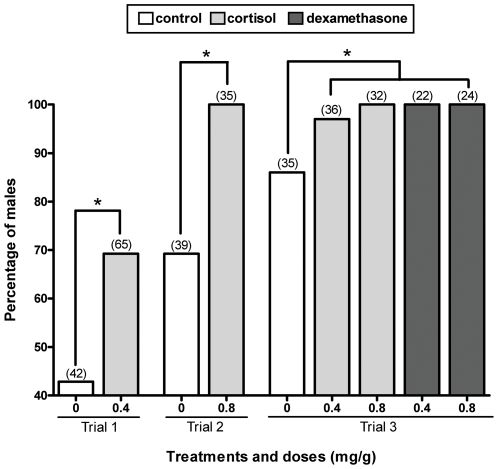
Percentage of males in groups treated with cortisol and the synthetic glucocorticoid receptor (GR) agonist Dexamethasone. All groups were reared at an intermediate temperature (24°C) during the trials. Groups treated with cortisol and Dexamethasone had increased proportions of males compared to the respective controls. Numbers within parenthesis indicate the sample number; asterisks indicate statistically significant differences between groups in the same trial (χ^2^; P≤0.05; GraphPad Prism v.4.00).

We then administered exogenous cortisol to larvae reared at the MixPT (24°C) between 1 and 10 wah to analyze its effects on key molecular events involved in the sex differentiation process of this species, e.g. the expression of *amh* and *cyp19a1a* mRNA [22]–[24] and gonadal apoptosis [25], and on the sex ratios. We also measured the whole-body concentrations of cortisol in treated larvae but this analysis failed to demonstrate a significant difference in comparison to the control group (data not shown), probably as a result of the relatively long time elapsed between the last feeding (evening) and sampling (morning) and the rapid cortisol clearance (see [10], [21], [26]). Nevertheless, the analysis of mRNA transcript abundance showed that all individuals from the group administered cortisol (0.8 mg/g; n = 10) presented a male-like pattern of *amh* upregulation and *cyp19a1a* downregulation at 6 wah (Fig. 2; see [22]–[24]). Likewise, the TUNEL assay revealed a higher incidence of apoptosis in the gonads of cortisol-treated fish compared to the controls on week 6 (Fig. 3). Almost all of the cells in the anterior sections of the right lobe of the gonad of these larvae were TUNEL-positive, a pattern typically associated with masculinization in this species [25]. These early molecular signs of masculinization were corroborated by the histological analysis conducted 2–3 months after cortisol withdrawal. Thus, in three independent trials, groups administered cortisol consistently had higher percentages of males compared to the respective controls and those with the highest dosage (0.8 mg/g) were all male (Fig. 4). Mortalities were negligible after the first week, when the fish started to ingest the cortisol-treated diets, and hence could not have caused the observed sex ratio deviations.

**Figure 5:**
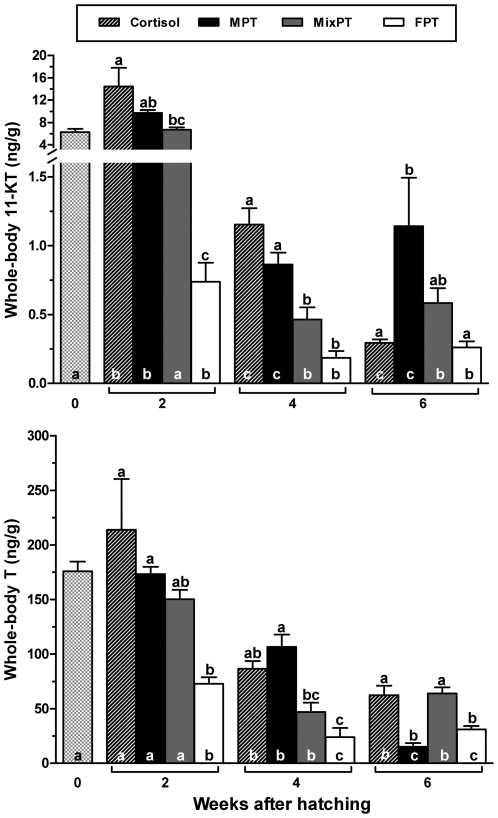
Concentrations of 11-ketotestosterone (11-KT) and testosterone (T) in cortisol-treated larvae reared at MixPT (24°C) and in untreated larvae reared at MPT (29°C), MixPT (24°C) and FPT (17°C) between 0 and 6 wah. Different letters inside and above the bars indicate statistically significant differences between weeks for the same temperature and between treatments on the same week, respectively (One-way ANOVA followed by the Tukey test; P≤0.05; GraphPad Prism v.4.00). Larvae fed cortisol showed higher titres of 11-KT than the corresponding control (MixPT) between 2 and 4 wah. Both 11-KT and T were significantly higher in the MPT compared to the FPT on the same weeks.

We then set out to obtain basic clues on the mechanism(s) behind cortisol-induced masculinization. As noted above, earlier studies have pointed out to a possible interference by cortisol in the biosynthesis of male steroids [12], [14]–[17] although the magnitude and even the direction of this interference is far from being consensual [16], [17], [27], [28]. We examined this possibility using the results of gene (*cyp19a1a*) expression and the measurement of the whole-body concentrations of 11-KT, the main fish androgen [15], [16], [29], and T in control larvae from the three thermal conditions (FPT, MixPT, and MPT) and in larvae administered exogenous cortisol (8 mg/g) at the MixPT. This analysis showed that larvae at the FPT had significantly less of these hormones than those at the MPT between 2 and 4 weeks and that the 11-KT and T titres at the MixPT and MPT were particularly high at 2 wah (Fig. 5), a time which appears particularly critical for sex determination in pejerrey at these temperatures [20]. Furthermore, cortisol administration was associated with significant increases in 11-KT, but not T, during the first 4 weeks. Thus, our results for pejerrey larvae do not support the view that elevated cortisol causes decreased 11-KT production through substrate competition for enzymes mediating both its metabolism an the synthesis of 11-KT [15], even though the sustained high concentrations of cortisol (see Fig. 1) conceivably placed a continued constraint on 11-KT biosynthesis. The reason for the unexpected 11-KT elevation remains unknown but it is unlikely to depend solely on the cortisol-induced downregulation of *cyp19a1a* transcription described previously because it was evident already at 2 wah and *cyp19a1a* expression at the MixPT only begins at 4 wah [23], [24]. The expression of *cyp19a1b* (brain aromatase) starts earlier (2 wah) but this enzyme is also unlikely to be involved because its expression is actually promoted by high temperatures [30]. It remains to be seen if cortisol can be converted into androgen(s) *in vivo* [12], [17] in sufficient amounts to explain our results.

Next, we examined whether glucocorticoid receptors (GRs), which are known to be responsible for the transcriptional regulation of a myriad of genes involved in cell differentiation, proliferation and apoptosis across cell types and tissues including the gonads [10], [16], [31]–[34] might also play a role in the observed masculinization. In this study, we administered the synthetic, non-metabolizable GR agonist Dexamethasone [10] to larvae reared at the MixPT and observed that the two dosages tested (0.4 and 0.8 mg/g) resulted in 100% masculinization, as seen with the highest dosage of cortisol (Fig. 4). This effect is not surprising given the stronger GR agonist potency of Dexamethasone compared to cortisol [10] and is a compelling evidence that cortisol might affect the expression of genes involved in sex differentiation at these early developmental stages. As noted previously, cortisol administration induced gonadal apoptosis, *amh* upregulation and *cyp19a1a* downregulation in pejerrey larvae. Glucocorticoid-induced apoptosis has been reported also in the rat testis [33] but we can not yet discriminate whether the above processes were directly induced by cortisol or whether they were a consequence of the ongoing masculinization. For instance, the former two could be simply the result of *cyp19a1a* regulation since estrogens are known to inhibit gonadal apoptosis in fish [35] and to induce *amh* downregulation in pejerrey larvae [23]. Moreover, the presence of a glucocorticoid-responsive element (GRE) in the pejerrey *cyp19a1a*, as reported in a sequential hermaphroditic fish [36] is yet to be verified. Thus, we are currently searching for other genes containing GREs that might help explain the early dimorphism in androgen content (see [10], [37]). This search has now been extended to genes expressed in the brain and pituitary, as their masculizination/feminization by temperature is suspected of setting the blueprint for gonadal differentiation in pejerrey [30], [38], [39]. Additional pathways for cortisol effects might include nongenomic actions [10], [37], and this possibility also needs to be examined.

Overall, the results of sex ratios with cortisol and Dexamethasone administration and all other analyzes performed in this study provide consistent evidence that cortisol may play a role during high temperature-induced testicular differentiation in pejerrey. The findings of this and other studies suggest multiple/complex pathways for cortisol action on sex differentiation including, but not limited to endocrine regulation, differential gene transcription, and apoptosis induction. Comprehensive studies are being undertaken to provide molecular and biochemical details on the relationship between high temperature, stress, cortisol, and sex determination as well as the implications of these findings for natural populations of pejerrey. It remains to be seen if wild pejerrey larvae will chose the stressful, “cortisol-rich” masculinizing high temperatures on their own. The ongoing studies also aim to clarify if there is any genetic basis for the individual variation in the cortisol titre observed at the MixPT and the association with gonadal fate at this temperature, and whether the low cortisol titre observed at the FPT is the key permissive element for feminization. Since other environmental disruptors of homeostasis (e.g. the social environment and salinity) may be also linked to elevated body cortisol and sex reversal [14], [15], [40], it will be interesting to examine whether the molecular and biochemical findings of this study apply also to other fish species with TSD and to other forms of ESD. These findings and recent reports of cortisol effects on sex determination of TSD reptiles [13], [41] and even in birds [42], which have GSD (though this probably involves a different mechanism), provide support to the notion that stress responses may be involved in various forms of environmental sex determination.

## Materials and Methods

### Experimental design, rearing conditions, and sampling procedures

Three experiments were conducted for examination of the effects of cortisol and a cortisol analogue administration on sex ratios and to obtain samples for the hormonal and molecular analyzes. Replicates could not be run because of the small number of eggs available for each experiment, which were obtained by pairing of one female and one male. Thus, experimental manipulation of the sex ratios with cortisol administration was performed three times for confirmation of the results. Fertilized eggs were obtained by artificial insemination and incubated in flowing water at 18–19°C until hatching. Newly hatched larvae were then transferred to 60 l tanks and reared at 17°C (FPT), 24°C (MixPT), or 29°C (MPT) for up to 18 weeks under a constant light cycle (16L-8D) and salinity of 0.2–0.4%. The tanks with larvae were equilibrated to the experimental temperatures within 12 hours, a protocol validated in previous studies that showed no significant effects of temperature acclimation rate on the response of sex ratios. Fish were fed powdered fish food (TetraMin flakes, Melle, Germany) to satiation 4–5 times daily from early morning and a meal of live *Artemia* nauplii in the evening. For the pharmacological treatments, conducted at the sexually neutral temperature of 24°C, tanks were initially stocked with 50–70 larvae, of which 22–65 remained at the end of the experiment. Mortalities were concentrated in the first days after stocking but a precise count could not be obtained due to the fast (overnight) decay of dead bodies. Dead larvae were rarely found after the first week, when the larvae had grown considerably larger and began to feed on the experimental diets. Experimental diets were incorporated with cortisol and Dexamethasone (0.4 mg and 0.8 mg/g of diet; both from Sigma, St. Louis, MO, USA). Sampling was performed at hatching and then biweekly between 1 and 7 wah for EIA assay of body cortisol (Table S1) and between 2 and 6 wah for 11-KT and T titres (Table S2), at 4, 6 and 7 wah depending on the rearing temperature for gene expression (n = 10), and 4, 5 and 6 wah for TUNEL analysis of gonadal cell apoptosis (n = 3–10). Eggs and larvae for hormone measurements were sampled either as individuals or pools of larval trunks, depending on the size of the fish (which varies with growth at different temperatures), in order to obtain a minimum of about 100 mg of tissue for each analysis (see Tables S1, S2). Samples for histological determination of sex ratios (n = 22–65 per group) were taken at the end of 18 weeks. The choice of sampling times took in consideration previous studies on the sex differentiation of this species [19], [20], [22]–[24]. Larvae were anesthetized in ice-water and immediately fixed/stored following the procedures outlined in the following sections.

### EIA measurement of body cortisol, 11-ketotestosterone and testosterone

EIA was performed using the Cortisol Express EIA Kit and the 11-ketotestosterone (11-KT) and Testosterone (T) EIA Kits according to the instructions from the manufacturer (Cayman Chemical, Ann Arbor, USA). These kits are highly specific for cortisol, 11-KT, and T, with less than 2% crossreactivity with cortisol's inactive form cortisone, T, and 11-KT, respectively, and have lower detection limits of 20, 1.3, and 6 pg/ml, respectively (manufacturer's information). Individual or pools of larval trunks (Tables S1, S2) were homogenized in PBS on ice and suspended in 2 ml of diethyl ether for 15 min at 4°C. Homogenates were then centrifuged at 2,500 rpm for 2 min and frozen at −80°C for 15–30 min to recover the diethyl ether-based liquid phase. This procedure was repeated three times. After evaporation of the diethyl ether under N_2_, the samples were immediately resuspended in EIA buffer and analyzed in a microplate reader (Biorad Model 550, Hercules, USA) following the kit instructions. The recovery rate was estimated by the cold-spike method to be >85% and the intra- and inter-assay variation (CV%) ranged from 4 to 13%. The significance of the differences between groups was determined by ANOVA followed by the Tukey test using GraphPad Prism (v.4.00; GraphPad Software, San Diego, CA, USA). Differences were considered as statistically significant at P≤0.05.

### Histological determination of sex ratios

Trunks of larvae collected at the end of the experimental period were fixed in Bouin's solution overnight and stored in 70% ethanol. Samples were subsequently dehydrated in an ascending ethanol series, embedded in Paraplast Plus (McCormick, St. Louis, USA), sectioned transversally at a thickness of 6 µm, and mounted on glass slides. Slides were stained with hematoxylin-eosin and observed under a microscope for determination of gonadal sex following criteria outlined in previous studies [19], [20]. The significance of the differences between groups was determined by the χ^2^ method using GraphPad Prism (v.4.00; GraphPad Software, San Diego, CA, USA). Differences were considered as statistically significant at P≤0.05.

### Relative Quantification Real Time RT-PCR analysis of amh and cyp19a1a mRNA

Samples were fixed in RNAlater and stored at −80°C until processing. The *amh* and *cyp19a1a* expressions in cortisol-treated individuals were determined and compared to threshold expression values for these genes during male and female differentiation. Threshold values were calculated as the mean+2SD of the *amh* and *cyp19a1a* expression values at the FPT (6 wah) and MPT (7 wah), respectively, following previous studies [22]–[24]. Total RNA was extracted from larval trunks using 1 ml of TRIZOL Reagent (Invitrogen) following the manufacturer's instructions. RNA samples (1 µg) were treated with Deoxyribonuclease I Amplification Grade (Invitrogen) and reverse-transcribed using SuperScript III RNase H- (Invitrogen) with oligo(dT)12–18 following the manufacturer's instructions. The primer sets were as described previously [23], [24]. Real Time PCR was performed in 20 µl reaction volumes containing 2x Power SYBR® Green PCR Master Mix (ABI), 1 µl of first strand cDNA (approximately 25 ng) and 5 pmol of each primer in a 7300 Real Time PCR System (Applied Biosystems, Foster City, USA). Transcript abundance was quantified using the Standard Curve Method and normalized against β-actin values (which in turn was not affected by the cortisol treatment (Table S3) or by temperature [23], [24]).

### TUNEL assay of gonadal apoptosis

Samples were fixed overnight in 4% paraformaldehyde, embedded in Paraplast Plus, and sectioned transversally at a thickness of 5 µm. Representative histological sections of the anterior portion of the gonad where chosen for analysis following the findings of previous studies [25]. Slides were incubated with 1 µg/mL Proteinase K (Invitrogen) for 10 min at 37°C, refixed in 4% paraformaldehyde for 10 min, and pre-incubated with terminal deoxynucleotidyl transferase (TdT) buffer for 10 min at 37°C. Laddered DNA labelling was carried out with 1 mM fluorescein-dUTP (Perkin Elmer, Boston, USA) and 40 units/mL TDT enzyme (Roche, Penzberg, Germany) in TdT buffer for 80 min at 37°C. Each TUNEL assay included a positive control slide that was incubated with 1 µg/mL DNase (Invitrogen) for 10 min at room-temperature and a negative control slide where the TDT enzyme was replaced with distilled water. Slides were counterstained with DAPI and observed under a fluorescence microscope (Eclipse E600 Nikon, Tokyo, Japan). Images were captured and digitalized with a CCD camera (Penguin 600CL, Pixera Corp, San Jose, CA, USA).

## Supporting Information

Table S1 Sample sizes for EIA measurement of whole-body cortisol
(0.06 MB DOC)

Table S2 Sample sizes for EIA measurement of whole-body 11-ketotestosterone and testosterone
(0.03 MB DOC)

Table S3 Ct values for β-actin in cortisol-treated and control larvae at 4 and 6 weeks after hatching.
(0.04 MB DOC)
