# Supplementary material for: Cortisol-Induced Masculinization: Does Thermal Stress Affect Gonadal Fate in Pejerrey, a Teleost Fish with Temperature-Dependent Sex Determination?
Source: PLoS One. 2009 Aug 7;4(8):e6548. doi: 10.1371/journal.pone.0006548 (PMC2717333; doi:10.1371/journal.pone.0006548)
Supplement: Table S1 — Sample sizes for EIA measurement of whole-body cortisol (0.06 MB DOC) [file pone.0006548.s001.doc]

Table S1. Sample sizes for EIA measurement of whole-body cortisol.

| Weeks after hatching | Number of pools (individuals/pool)  FPT MixPT MPT | | |
| --- | --- | --- | --- |
| 0 | 3 (40) | | |
| 1 | 2 (25) | 2 (20) | 2 (20) |
| 3 | 2 (10) | 4 (5) | 4 (5) |
| 5 | 3 (3) | 7 (1) | 3 (2) |
| 7 | 2 (1) | 5 (1) | 6 (1) |

Table SII. Sample sizes for EIA measurement of whole-body 11-ketotestosterone and testosterone.

| Weeks after hatching | Number of pools (individuals/pool)  FPT MixPT MixPT+ MPT  Cortisol | | | |
| --- | --- | --- | --- | --- |
| 0 | 3 (40) | | | |
| 2 | 2 (15) | 2 (5) | 2 (5) | 4 (5) |
| 4 | 2 (5) | 9 (1) | 3 (2) | 5 (2) |
| 6 | 2 (2) | 7 (1) | 5 (1) | 3 (2) |

Table SII. Ct values for β-actin in cortisol-treated and control larvae at 4 and 6 weeks after hatching.

| Group | 4 weeks after hatching | | 6 weeks after hatching | |
| --- | --- | --- | --- | --- |
|  | cortisol | control | cortisol | control |
| β-actin  Ct values | 18.69 | 18.26 | 18.78 | 20.89 |
|  | 18.34 | 16.96 | 19.44 | 20.37 |
|  | 17.8 | 18.67 | 18.01 | 19.26 |
|  | 17.61 | 21.24 | 20.14 | 19.52 |
|  | 19.13 | 17.09 | 17.94 | 19.45 |
|  | 18.92 | 21.84 | 17.74 | 18.16 |
|  | 19.01 | 18.82 | 23.67 | 19.38 |
|  | 20.33 | 18.82 | 18.60 | 18.63 |
|  | 19.94 | 21.62 | 19.58 | 21.00 |
|  | 20.66 | 17.96 | 19.81 | 19.12 |
| mean±SD | 19.04±1.02* | 19.13±1.81 | 19.37±1.73* | 19.58±0.92 |

***No statistical difference with the control group (p>0.05; ANOVA test).
