# Supplementary material for: Cortisol-Induced Masculinization: Does Thermal Stress Affect Gonadal Fate in Pejerrey, a Teleost Fish with Temperature-Dependent Sex Determination?
Source: PLoS One. 2009 Aug 7;4(8):e6548. doi: 10.1371/journal.pone.0006548 (PMC2717333; doi:10.1371/journal.pone.0006548)
Supplement: Table S2 — Sample sizes for EIA measurement of whole-body 11-ketotestosterone and testosterone (0.03 MB DOC) [file pone.0006548.s002.doc]

Table S2. Sample sizes for EIA measurement of whole-body 11-ketotestosterone and testosterone.

| Weeks after hatching | Number of pools (individuals/pool)  FPT MixPT MixPT+ MPT  Cortisol | | | |
| --- | --- | --- | --- | --- |
| 0 | 3 (40) | | | |
| 2 | 2 (15) | 2 (5) | 2 (5) | 4 (5) |
| 4 | 2 (5) | 9 (1) | 3 (2) | 5 (2) |
| 6 | 2 (2) | 7 (1) | 5 (1) | 3 (2) |
